# Pre-notification letter type and response rate to a postal survey among women who have recently given birth

**DOI:** 10.1186/s12874-015-0097-8

**Published:** 2015-12-01

**Authors:** Angela L. Todd, Maree Porter, Jennifer L. Williamson, Jillian A. Patterson, Christine L. Roberts

**Affiliations:** Clinical and Population Perinatal Health Research, Kolling Institute, University of Sydney, Northern Sydney Local Health District, St Leonards, New South Wales, 2065 Australia; Sydney Medical School Northern, University of Sydney, St Leonards, New South Wales 2065 Australia

**Keywords:** Survey research, Response rate, Patient satisfaction, Record linkage, Randomised controlled trial

## Abstract

**Background:**

Surveys are commonly used in health research to assess patient satisfaction with hospital care. Achieving an adequate response rate, in the face of declining trends over time, threatens the quality and reliability of survey results. This paper evaluates a strategy to increase the response rate in a postal satisfaction survey with women who had recently given birth.

**Methods:**

A sample of 2048 Australian women who had recently given birth at seven maternity units in New South Wales were invited to participate in a postal survey about their recent experiences with maternity care. The study design included a randomised controlled trial that tested two types of pre-notification letter (with or without the option of opting out of the survey). The study also explored the acceptability of a request for consent to link survey data with existing routinely collected health data (omitting the latter data items from the survey reduced survey length and participant burden). This consent was requested of all women.

**Results:**

The survey had an overall response rate of 46 % (913 completed surveys returned, total sample 1989). Women receiving the pre-notification letter with the option of opting out of the survey were more likely to actively decline to participate than women receiving the letter without this option, although the overall numbers of women declining were small (27 versus 12). Letter type was not significantly associated with the return of a completed survey. Among women who completed the survey, 97 % gave consent to link their survey data with existing health data.

**Conclusions:**

The two types of pre-notification letters used in our study did not influence the survey response rate. However, seeking consent for record linkage was highly acceptable to women who completed the survey, and represents an important strategy to add to the arsenal for designing and implementing effective surveys. In addition to aspects of survey design, future research should explore how to more effectively influence personal constructs that contribute to the decision to participate in surveys.

## Background

Surveys are commonly used in health research to assess patient satisfaction with hospital care. Many countries conduct large population-based surveys, for example, The Commonwealth Fund in the United States of America (USA) conducts both national and international comparisons of patients’ reported hospital care experiences [1]; and The National Health Service (NHS) in the United Kingdom (UK) also regularly collects information from patients about the health care they receive [2].

The most common reason that women are admitted to hospital is for care during pregnancy and birth [3]. Maternity patients are notably different from general hospital populations: they are comparatively young, healthy and usually attend hospital for a relatively short time. For most, the outcome of their stay is very positive: they leave with a healthy newborn. A number of large-scale maternity-specific surveys have been conducted to assess satisfaction with care in the US [4–6], Canada [5, 7], the UK [8–10], and Australia [11–18]. The majority have been conducted by post [8, 10, 16–18], and while satisfaction levels have been high, response rates have varied from 30–71 %. Three of the surveys have combined postal surveys with an online version, however uptake of the latter option was comparatively low, ranging from 7–16 % of respondents [8, 15, 16].

Postal surveys represent a cost-efficient method for data collection. Achieving an adequate response rate has traditionally been viewed as an indicator of data quality and reliability [19]. Evidence suggests survey response rates have been declining steadily over time [20–22]. Non-response represents a combination of refusal to participate and non-contact with targeted participants. Non-contact, usually due to residential mobility, is considered a much larger component of survey non-response than typically acknowledged [23]. Population-based estimates suggest 25–35 % of people will change residence over a two to three year period [24, 25], and over 40 % in five years [26].

Various strategies and factors have been shown to be effective in increasing response rates to surveys. A Cochrane review identified 481 randomised controlled trials that tested 110 different ways of increasing response rates to postal surveys [27]. More effective strategies included: monetary incentives; shorter rather than longer surveys; surveys that addressed topics salient to the responder; avoidance of sensitive questions; assurance of confidentiality; pre-notification letters; reminders; providing a second copy of the survey at follow-up; personalised letters/surveys; and a university as the survey sponsor (rather than a government agency or commercial organisation).

Subsequent to this review, a Swedish trial with parents of young children tested the effect of different types of initial contact letters on survey consent and response rates. The results showed that mailing a pre-notification letter with a consent form, or a pre-notification letter with an opt out option for the survey yielded significantly higher response rates than directly sending the survey without any pre-notification (61 % and 72 % versus 47 % respectively) [28]. The authors suggest mothers and fathers who received the opt out option but did not take it may have felt a certain obligation to respond, resulting in a higher response rate. The Cochrane review above identified four prior surveys that also included an opt out option, and reported mixed results in terms of effect on response rates [29]. Thus, the effectiveness of this strategy merits further investigation.

Linking health survey data with existing data sets is an efficient and cost-effective strategy to widen the range of information available and the research questions that can be answered. In some instances it can also have the advantage of reducing the length of a survey and participant burden, by accessing the required information from other sources. Record linkage of survey data with hospital and mortality databases has been used to examine longitudinal patterns of health, illness and disease [30–32]. Consent for linkage from study participants has varied (74–96 %), with some evidence of lower rates of consent associated with younger age, lower education levels, lower socioeconomic status and ethnic minority groups [33–35]. Consent for record linkage has not been used in past maternity health surveys, but has been requested of mothers to link survey data with their children’s birth records [33, 34].

### Aims and objectives

In this paper we report on methodological aspects of a postal survey among women who had recently given birth. While the broader objectives of the survey study were to capture women’s expectations of, and experiences with maternity care, and to explore whether maternal and birth characteristics are associated with those experiences, we also planned two methodological studies. One was to assess the quality of the survey tool that was developed [36], and the second, reported in this paper, was to examine a strategy intended to increase the survey response rate: a pre-notification letter with an opt out option (compared to a letter without this option).

## Methods

### Survey participants

New South Wales (NSW) is the largest state in Australia by population, with more than 7 million people. NSW accounts for one in three births nationally, representing around 100,000 births per year. Australian maternity care includes both public and private care; all women are covered by national health insurance which provides maternity care at no cost for public patients in public hospitals, but about one-third of women have private medical insurance or pay for private obstetric care, which can take place at a public or private hospital.

In the present study, all women who gave birth between 1 May and 31 July 2013 at seven public maternity units in two neighbouring health districts in NSW were eligible to participate (estimated to be approximately 2000 women). These seven units account for approximately 11 % of births in public hospitals in NSW, and represent a mixture of urban and regional, and tertiary and smaller health services. Women giving birth were identified from each maternity unit’s clinical obstetric database (a uniform system among all units), which records personal details and clinical information about women’s pregnancy and birth. While other maternity surveys have excluded women who have had a stillbirth or early neonatal death [8, 11, 13, 17, 18], in this study all women were given the same opportunity to participate or decline; there were no a priori exclusions. The study was approved by the NSW Population & Health Services Research Ethics Committee (HREC/12/CIPHS/82).

### Survey design

A survey instrument was developed, drawing on questions used in previous maternity surveys [8, 13, 16], and consultations with stakeholders – obstetricians, midwives, consumer representatives, health service administrators, policy-makers, survey design experts and perinatal researchers. After pilot testing, the final version was structured around the three main maternity periods (antenatal, birth, and postnatal), and addressed topics such as: satisfaction with care; responsiveness and communication with health care providers; the extent to which women’s expectations and desires were met; and their involvement in decision-making about their care. The survey comprised 123 questions and took approximately 20–30 min to complete. Further details are available elsewhere [37].

Consent to participate in the study was implied by the return of a completed survey, however the survey sought written consent from each woman to link her survey responses with health information recorded in each maternity unit’s clinical obstetric database. Validation studies have shown details of labour and birth are reliably reported in routinely collected perinatal data collections in New South Wales [38, 39]. We identified 46 data items in the clinical obstetric database of potential relevance to our overall research study. They included pre-existing medical conditions, pregnancy-related complications, labour and birth details (for example, indications for obstetric interventions) and infant outcomes (such as Apgar scores at 1 and 5 min). This information was not collected in the survey. Women refusing consent to record linkage were still eligible to complete the survey. Information about the request for consent was included in a participant information pamphlet that was sent to women with the survey. The wording of the request for consent on the survey form was:

*I consent to take part in this survey, and have my survey responses linked to health information about me and my baby’s birth, previously recorded by the hospital where I gave birth.*

### Survey methodology

Approximately 3–4 months after giving birth, eligible women were mailed a personalised pre-notification letter (with or without the option of opting out of the survey). Women were randomly allocated to receive one of two letters: *Actively Decline* (the letter gave information about the survey and instructions for how to withdraw from the study and not receive the survey), or *No Action* (the letter gave information about the survey only). The only difference between the two letters was the inclusion of the following paragraph in the *Actively Decline* letter:

*If you prefer not to complete the survey, please contact (staff person’s name) within 7 days, quoting your study reference number, and she will remove your name from the survey group. You can call (staff person’s name) on (telephone number), send an email to (email address), or write to her at (mailing address).*

Letter allocation was 1:1, determined by computer randomisation using random number generation, and stratified by hospital. Two to three weeks after the pre-notification letter was distributed, a survey package was sent to all women, except those who had actively declined to participate or whose letter had been returned as undeliverable (e.g., no longer at the same address). The survey package contained a short personalised cover letter, participant information pamphlet, the survey, and a reply paid envelope for return of the survey. The cover letter indicated that women who did not wish to participate could return a blank survey. Each woman was assigned a unique study number to facilitate record linkage; this number appeared on the survey with no other identifying details. A reminder letter was sent approximately three to four weeks later to women who had not responded. Thus women could receive up to 3 contacts about the survey. All correspondence with the women was by mail (although women could withdraw from the study by mail, email or telephone).

The survey and associated mailing protocol included a large number of features that have been shown to increase response rates: personalised pre-notification letter; survey topic that was highly salient to participants; attractive survey design; postage-paid return envelope; 2–3 contacts with participants; survey sponsored by an academic organisation; and assurances of data confidentiality and anonymity [29].

Considerable effort was made to protect women’s identifying information and conform with state privacy legislation. The data manager of the clinical obstetric database was responsible for sample identification, assignment of the unique study number, and extraction of personal and health data from the clinical obstetric database. A person not otherwise involved in the study coordinated the mail-out of pre-notification letters, the survey packages and the reminder letters. This person had access to women’s names and addresses but not their health or survey information. All completed surveys were received by the researchers, containing each woman’s unique study number but no other identifying details. At the close of the survey, the person coordinating the mail-out activities provided the data manager with two lists of unique study numbers - for the survey respondents, and for the remaining women (‘no response’). The data manager provided the researchers with a data extract from the clinical obstetric database for the approved data items for those women who had given consent to link their survey responses with their health data. The unique study number was then used by the researchers to merge the two data sources. The extracted health information did not include any personal identifiers other than the unique study number. The data manager generated aggregate descriptive statistics for the ‘no response’ women, to allow for testing of sample bias.

### Statistical analyses

The survey response rate was calculated using as the denominator the total mail-out number less the number of letters returned as undeliverable [40]. We tested for response bias by comparing survey respondents with the remaining women (combining women who declined and women who did not return a survey) on available maternal and obstetric data recorded during pregnancy and birth. We also used logistic regression to assess the odds of responding to the survey for each of the maternal and obstetric factors available, and to explore the potential effect modification of letter type. We defined a woman’s socio-economic status using the Australian Index of Relative Socioeconomic Advantage/Disadvantage (IRSAD) based on her residential post code [41]. The most disadvantaged women were defined as those with post codes corresponding with the post codes of the lowest 20 % of the NSW population, and the most advantaged women were those with post codes corresponding with those of the highest 20 % of the population. We tested whether the type of pre-notification letter (*Actively Decline, No Action*) a woman received had any effect on the decision to respond to the survey. Finally, within the survey respondent group, we compared the women who consented to linkage of their survey with clinical health data versus those who did not. Descriptive statistics, Fisher’s exact test, Chi-squared tests, contingency table analysis and non-parametric tests were used as appropriate, with a p-value of 0.05 considered statistically significant. All analyses were performed using SAS 9.3 (SAS Institute, Cary NC, USA).

### Power calculations

The randomised trial of the two types of pre-notification letter with a sample of approximately 2000 women would allow us to detect, with 90 % power, an absolute increase in response rate of 7 % from a baseline rate anywhere between 35 % and 70 % (response relative risks of 1.20 and 1.10 respectively).

## Results

**Fig. 1:**
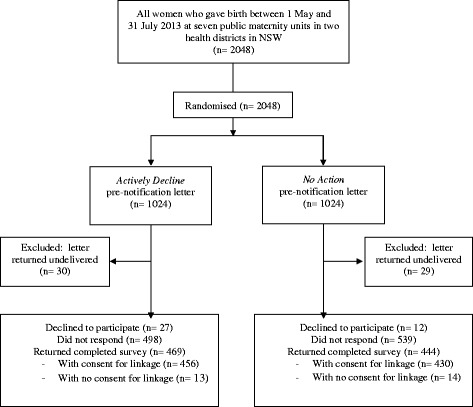
Flow diagram for postal survey response rate

A total of 2048 women gave birth between 1 May and 31 July 2013 at the public maternity units in the two participating health districts, and were eligible to participate in the survey. Following randomisation, 1024 women received the *Actively Decline* pre-notification letter, and 1024 women received the *No Action* pre-notification letter. Fifty–nine letters (3 %) were returned as undelivered, leaving a sample of 1989 (Fig. 1). Available maternal, obstetric and newborn details for the 1989 women, recorded by the maternity units at the time of pregnancy and birth, are shown in Table 1. Approximately 64 % of women were aged 30 years or more, 60 % were born in Australia, 43 % lived in areas of highest socio-economic advantage, 7 % reported smoking during pregnancy, 46 % were nulliparous, 70 % had a vaginal birth and 7 % had a preterm birth (<37 weeks).

**Table 1:** Maternal and newborn characteristics for all women, and respondents versus no survey returned

Characteristics	Study cohort	Respondents	No survey	*p*-value*	Univariate odds ratio
	*N* = 1989 (%)	*N* = 913 (%)	*N* = 1076 (%)		(95 % CI)^a^
Maternal age (years)					
≤24	233 (11.7)	66 (7.2)	167 (15.5)	*p* < 0.0001	0.50 (0.36–0.71)
25–29	487 (24.5)	214 (23.4)	273 (25.4)		Reference
30–34	750 (37.7)	362 (39.7)	388 (36.1)		1.19 (0.95–1.50)
≥35	519 (26.1)	271 (29.7)	248 (23.1)		1.39 (1.09–1.79)
Country of birth					
Australia	1196 (60.1)	559 (61.2)	637 (59.2)	*p* = 0.4	Reference
Other	793 (39.9)	354 (38.8)	439 (40.8)		0.92 (0.77–1.10)
Socio-economic index					
1 (lowest)	107 (5.4)	30 (3.3)	77 (7.2)	*p* < 0.0001	0.34 (0.22–0.53)
2	395 (19.9)	142 (15.6)	253 (23.5)		0.49 (0.39–0.63)
3	169 (8.5)	66 (7.2)	103 (9.6)		0.56 (0.40–0.79)
4	463 (23.3)	220 (24.1)	243 (22.6)		0.80 (0.64–1.00)
5 (highest)	855 (43.0)	455 (49.8)	400 (37.2)		Reference
Maternity unit					
1	37 (1.9)	16 (1.8)	21 (2.0)	*p* < 0.0001	1.37 (0.70–2.67)
2	674 (33.9)	241 (26.4)	433 (40.2)		Reference
3	585 (29.4)	280 (30.7)	305 (28.4)		1.65 (1.32–2.07)
4	29 (1.5)	17 (1.9)	12 (1.1)		2.55 (1.20–5.42)
5	245 (12.3)	133 (14.6)	112 (10.4)		2.13 (1.59–2.87)
6	149 (7.5)	75 (8.2)	74 (6.9)		1.82 (1.27–2.60)
7	270 (13.6)	151 (16.5)	119 (11.1)		2.28 (1.71–3.04)
Parity					
Nulliparous	923 (46.4)	460 (50.4)	463 (43.0)	*p* = 0.001	1.34 (1.13–1.61)
Multiparous	1066 (53.6)	453 (49.6)	613 (57.0)		Reference
Maternal smoking in pregnancy	140 (7.0)	25 (2.7)	115 (10.7)	*p* < 0.0001	0.24 (0.15–0.37)
Maternal BMI					
≤25	1283 (64.5)	609 (66.7)	674 (62.6)	*p* = 0.1	Reference
>25, ≤ 30	438 (22.0)	193 (21.1)	245 (22.7)		0.87 (0.70–1.10)
>30	268 (13.5)	111 (12.2)	157 (14.6)		0.78 (0.60–1.02)
Multiple pregnancy	28 (1.4)	13 (1.4)	15 (1.4)	*p* = 1.0	1.02 (0.48–2.16)
Mode of birth					
Vaginal	1148 (57.7)	530 (58.1)	618 (57.5)	*p* = 0.1	Reference
Instrumental	244 (12.3)	126 (13.8)	118 (11.0)		1.25 (0.94–1.64)
Pre-labour caesarean	309 (15.5)	128 (14.0)	181 (16.8)		0.95 (0.73–1.23)
Intrapartum caesarean	288 (14.5)	129 (14.1)	159 (14.8)		0.83 (0.64–1.06)
Postnatal length of stay (average days)	2.4	2.4	2.4	*p* = 1.0	-
Gestational age at birth (completed weeks)					
<37	136 (6.8)	61 (6.6)	75 (7.0)	*p* = 0.8	0.96 (0.67–1.36)
≥37	1853 (93.2)	852 (93.4)	1001 (93.1)		Reference
Infant gender					
Male	1024 (51.5)	456 (50.0)	568 (52.8)	*p* = 0.2	0.89 (0.75–1.07)
Female	965 (48.5)	457 (50.1)	508 (47.2)		Reference
Infant admission to intensive care or special care nursery	324 (16.4)	132 (14.6)	192 (18.0)	*p* = 0.04	0.78 (0.61–0.99)

*Chi-square test of comparison between Respondents and No Survey groups

^a^Odds ratios for Respondents versus No Survey

### Response rate and response bias

A total of 913 women returned a completed survey, representing a response rate of 46 %; 35 % of surveys were returned prior to receipt of a reminder letter, and 11 % following the reminder letter. Comparisons between the survey respondents (*n* = 913) and the remaining women (‘no survey’, *n* = 1076) using available maternal, obstetric and newborn details showed significant differences in maternal age (*p* < 0.0001), socio-economic status (*p* < 0.0001), maternity unit (*p* < 0.0001), parity (*p* = 0.001) smoking status (*p* < 0.0001), and infant admission to neonatal intensive care or special care nursery (*p* = 0.04) (Table 1). The odds of responding to the survey were higher if a woman was ≥35 years old compared to 25–29 years (Odds ratio (OR) 1.39, 95 % Confidence Interval (CI) 1.09–1.79), and differed by maternity unit attended (Table 1). The odds of responding were lower if a woman: was ≤24 years old compared to 25–29 years (OR 0.50, CI 0.36–0.71); lived in an area of socio-economic disadvantage compared to socio-economic advantage (OR 0.34, CI 0.22–0.53); smoked during pregnancy (OR 0.24, CI 0.15–0.37); and had a baby admitted to neonatal intensive care of special care nursery (OR 0.78, CI 0.61–0.99) (Table 1). The proportion of women experiencing a stillbirth or neonatal death was similar in the two groups although the numbers were small (respondents = 7 (0.8 %) versus no survey = 13 (1.2 %)).

### Effect of pre-notification letters

Of the sample of 1989 women, 994 received the *Actively Decline* letter and 995 the *No Action* letter. Letter type was not significantly associated with the decision to return a completed survey (*Actively Decline* = 469 (47 %, OR 1.11, CI 0.93–1.32, *p* = 0.25) versus *No Action* = 444 (45 %). However, two significant interactions were found for letter type by maternal age (*p* = 0.04), and letter type by BMI (*p* < 0.001). Women ≤24 years old, and women with a BMI >30 were twice as likely to complete the survey if they received the *Actively Decline* letter than the *No Action* letter (OR 2.20, CI 1.22–3.96; OR 2.05, CI 1.25–3.35 respectively) (Table 2).

**Table 2:** Effect of letter type on survey completion by maternal characteristics

Maternal characteristic	Odds ratio	95 % confidence interval	*p*-value
Maternal age (years)			
≤24	2.20	1.22–3.96	
25–29	1.30	0.91–1.86	0.04
30–34	0.99	0.74–1.31	
≥35	0.88	0.62–1.24	
Country of birth			
Australia	1.06	0.84–1.33	0.54
Other	1.19	0.90–1.57	
Socio-economic index			
1 (lowest)	1.1	0.5–2.5	0.06
2	1.9	1.2–2.9	
3	0.7	0.4–1.3	
4	1	0.7–1.4	
5 (highest)	1.1	0.8–1.4	
Maternity unit	1.5	0.4–5.7	
1	1.33	0.97–1.82	0.11
2	0.99	0.71–1.36	
3	0.30	0.06–1.50	
4	1.85	1.11–3.09	
5	1.14	0.60–2.17	
6	0.78	0.48–1.26	
7	1.52	0.40–5.71	
Parity			
Nulliparous	1.15	0.89–1.49	
Multiparous	1.07	0.84–1.37	0.69
Maternal smoking in pregnancy			
No	1.11	0.93–1.33	0.59
Yes	1.42	0.59–3.43	
Maternal BMI			
≤25	1.20	0.96–1.49	
>25, ≤ 30	0.62	0.42–0.90	**<0.001**
>30	2.05	1.25–3.35	
Multiple pregnancy			
No	1.11	0.93–1.33	
Yes	1.02	0.23–4.53	0.91
Mode of birth			
Vaginal	1.02	0.81–1.28	0.67
Instrumental	1.26	0.76–2.09	
Pre-labour caesarean	1.29	0.82–2.05	
Intrapartum caesarean	1.27	0.80–2.02	
Gestational age at birth (completed weeks)			
<37	1.26	0.64–2.48	
≥37	1.10	0.92–1.32	0.70
Infant gender			
Male	1.05	0.81–1.34	
Female	1.17	0.91–1.52	0.52
Infant admission to intensive care or special care nursery			
No	1.11	0.92–1.35	0.83
Yes	1.06	0.68–1.65	

As expected, women receiving the *Actively Decline* letter were more likely to decline to participate than women receiving the *No Action* letter, although the overall numbers were small (*n* = 27 versus *n* = 12, *p* = 0.02, Table 3).

**Table 3:** Type of pre-notification letter for respondents, women who declined and women who did not respond

	Pre-notification letter		
	Actively Decline N (%)	No Action N (%)	Total
Respondents	469 (47.2)	444 (44.6)	913
Women who declined	27 (2.7)	12 (1.2)^a^	39
Women who did not respond	498 (50.1)	539 (54.2)	1037

^a^ *Actively Decline* versus *No Action*: *χ* ^2^ = 5.90, df = 1, *p* = 0.02

### Effect of request for consent to link data

Among the 913 women who returned a completed survey, 886 (97 %) consented to linkage of their survey and health data, and 27 (3 %) did not. Comparisons between these two groups using available maternal, obstetric and newborn details showed no significant differences due to the small number of non-consenters.

## Discussion

We achieved a response rate of 46 % in a postal survey to Australian women who had recently given birth. Women who were younger, living in areas of socio-economic disadvantage, and who smoked during pregnancy were less likely to return a completed survey, characteristics that have been shown to be inter-related [42]. Like our study, other maternity surveys have reported under-representation of younger and socially disadvantaged women [8, 16, 43]. Others have also reported lower survey participation rates among persons who engage in risk behaviours such as smoking, alcohol or drug use [22]. Clearly, there are difficulties in effectively engaging potential survey participants with these demographic characteristics, and different or supplementary strategies may be required to involve such groups in population studies. Some have suggested over-sampling techniques, financial incentives or mixed survey study designs (that combine mail, internet, telephone and/or face-to-face interviews) to encourage participation and minimise nonresponse bias from ‘difficult-to-reach’ populations [22, 44]. Social media may also offer some help in reaching younger adults, although the effectiveness of this option is yet to be well evaluated, and would be of limited value in studies such as the present one that sought to reach specific pre-selected individuals. Analytical tools may be used after the fact to weight and adjust survey samples for such under-representation [44], noting that some caution is needed since such techniques involve assumptions about the likely response patterns of non-respondents [45].

We incorporated a number of features in our survey design and methods known to increase response rates, as well as two additional strategies not used previously in maternity surveys. We tested a pre-notification letter that gave women the option of actively opting out of the survey, modeled on a similar approach in a Swedish study that yielded a higher response rate [28]. In the Swedish study, 19 % of parents who received the opt out option declined to participate in the survey, 71 % returned a survey and 9 % did not respond. In our study, only 3 % of women who received this letter type declined to participate, 47 % returned a completed survey and 50 % did not respond. Furthermore, in our study similar proportions of women in the two pre-notification letter groups returned a completed survey (47 % and 45 %), although there was some evidence that younger women and obese women were more likely to complete the survey if they received a pre-notification letter with an active opt out option. Unlike the Swedish study, we did not include a third group that received no pre-notification letter, so it is not possible to determine whether either type of pre-notification letter was more effective than no letter at all, although other evidence suggests this is likely [27]. In the Swedish study, it was suggested that if parents were presented with the opportunity to withdraw from a study and did not take it, they might feel more obligated to participate. This might explain the interaction result we found among the younger women who completed the survey in our study, although it is less clear whether this can account for the effect among the obese women. Perhaps the wording of the letter in our study did not engender the same feelings of obligation among the majority of women, or perhaps Australian women are less likely to respond in this way. The comparatively high proportion of women in our study who did not respond to the survey in any way suggests other factors may be at play.

Another strategy we incorporated in our survey design to promote participation in the survey was to seek consent from women to link their survey data with existing routinely collected data. This approach has not been used previously in maternity surveys, although it has been used in other health-related research [30–35]. The information provided to women in our survey package explained that the purpose of this request for consent was to avoid collecting duplicate data that already exist, reduce survey length and participant burden. The vast majority of women who returned a completed survey gave their consent to record linkage (97 %). The women’s consent allowed us to access 46 data items from a clinical obstetric database. Some of the items were unlikely to be known to most women and/or open to recall error. Had we included the 46 items in the survey, we estimate the length and time to complete the survey would have increased by about one-third, which may have negatively affected some women’s decision to participate. However, we do not know whether this request for consent dissuaded some women from responding to the survey at all. Testing this question in the present study, for example by randomising women to receive a survey with or without a request for consent to record linkage, was not practical, as the supply of survey data without the associated health information about a woman’s pregnancy and birth would be of limited value to the aims of this study. However, future research examining the contribution of a request for consent to record linkage on survey participation would be valuable, particularly among younger and economically disadvantaged participants who appear less likely to participate in surveys and less likely to give consent to linkage.

No response was received from just over half of the women selected for the survey sample. Some have suggested that residential mobility accounts for a significant proportion of survey non-response [23]. In our study, only a small proportion of letters (3 %) was returned as undeliverable, consistent with results reported in other maternity surveys (1.5–6 % [8, 13, 15–18]). However, other evidence suggests relatively high levels of residential mobility among women during pregnancy and following birth, for example, one study found 19 % of women having a first birth had moved between their pregnancy and 12 months postpartum [46]. In the present study, responses to a survey question about residential mobility indicated that 36 % of women had moved to a new location in the 12 months preceding the birth. It is therefore highly possible that a significant proportion of the non-responders to the survey had not been reached because they had also changed address.

Two theoretical models have been suggested to help understand why people participate in surveys or not [47–49]. One, drawing on social exchange theory, suggests that the decision to participate is a reasoned and calculated decision based on weighing perceived costs (e.g., time, privacy) and benefits (e.g., rewards, incentives, altruistic needs). The second model views the decision as more of a psychological process influenced by personal factors such as compliance with requests, helping tendencies, and social responsibility. It has been argued that respondent factors like these are likely to be more influential in the survey participation decision than survey design factors. As such, addressing the former is more likely to help increase response rates [48]. In the present study, women were specifically chosen by their date of giving birth; correspondence was addressed to them personally by name; the survey was designed to allow each women to tell ‘her story’ about her pregnancy and birth experiences; and the overall aim of the survey was to understand each woman’s maternity care experiences and improve services for other women in the future. Arguably, these features should have been highly salient to women and communicated the importance of their participation. However, the survey demanded time from women in the early months of their newborn’s life when women are often tired and possibly returning to paid work. Perhaps the time cost was too high for some women: rather than take steps to actively opt out of the survey or complete the survey, the easiest option was to do nothing.

In the present study, we included one reminder only and did not include another copy of the survey (for cost reasons). Of our 46 % response rate, 11 % (nearly a quarter of the responses) was obtained after the reminder letter. Other research has shown that a first reminder can increase survey response rates by between 10 % and 30 % [27, 50–53], and a second reminder by around 6–9 % [52, 53]. Inclusion of another survey with a reminder has been shown to have only a modest effect on response rates, of about 4–5 % [27, 50]. Nonetheless, if time and funds permit, survey researchers may benefit from using these additional strategies.

We developed a methodology for this study that allowed us to obtain identifying information about the selected sample for mailing purposes, and that also protected the identity of the individual women involved in the research project. This is often an area of focus for institutional review boards and independent ethics committees considering survey-based proposals. The request for consent from the women to access and use their previously recorded health information for linkage with their survey responses is also an ethical issue: we considered it unreasonable to ask women for a range of information about their pregnancy and birth experiences when it already exists. Future research could explore whether this view is shared by maternity care patients and other health care consumers. Information privacy and access rules vary across countries and jurisdictions, however in environments where such access is possible, it should be pursued. Not only does this strategy reduce the burden on survey participants in terms of data collection, but it is also likely to yield more accurate information. The very high consent rate in the present study suggests this is highly acceptable to many women.

Our response rate of 46 % is consistent with the results reported by several others who have conducted postal surveys with women who have recently given birth [8–10, 16, 17]. However, it was lower than we expected, despite using a range of strategies that have been shown to increase postal survey response rates [27]. Nonetheless, the value of a survey lies not only in its response rate; it depends also on the quality of the information obtained. Analysing and reporting the results of our survey to better understand women’s experiences with maternity care, to explore whether satisfaction varies with different clinical experiences, and to identify opportunities for health service improvements are our next focus.

## Conclusions

Maximising response rates is a common goal in survey projects, and is becoming more challenging in the face of declining trends over time. Giving survey participants an early opt out option did not increase response rates in this survey study. However, seeking consent for record linkage appeared highly acceptable and should be considered in survey design, given the benefits of using previously collected data, and reducing survey length and participant burden.

## Abbreviation

NSW: New South Wales (state of Australia)
